# Complete intraureteral stent placement relieves daytime urinary frequency compared with conventional placement in patients with an indwelling ureteral stent: post-hoc analysis of a randomized, controlled trial

**DOI:** 10.1038/s41598-020-72937-0

**Published:** 2020-09-28

**Authors:** Tomoaki Matsuzaki, Takashi Yoshida, Takashi Murota, Kazuyoshi Nakao, Makoto Taguchi, Hidefumi Kinoshita, Tadashi Matsuda

**Affiliations:** 1 Department of Urology and Andrology, Kansai Medical University, 2-3-1 Shinmachi, Hirakata, Osaka 573-1191 Japan; 2 Department of Urology and Andrology, Kori Hospital, Kansai Medical University, Osaka, Japan; 3 Department of Urology and Andrology, General Medical Center, Kansai Medical University, Osaka, Japan

**Keywords:** Medical research, Signs and symptoms, Urology

## Abstract

A previous randomized, controlled trial had demonstrated that complete intraureteral stent placement (CIU-SP) was superior to conventional stent placement (C-SP) in terms of improvement of stent-related urinary symptoms. However, it is unclear as to which subdomain symptom and cohort could benefit the most from CIU-SP compared to C-SP in urinary symptoms while considering the baseline urinary status. To determine this, a post-hoc analysis was performed using data from a previous study (CIU-SP group, n = 39; C-SP group, n = 41). We assessed the mean changes in the International Prostate Symptom Score (I-PSS) and the Overactive Bladder Symptom Score (OABSS) from baseline to day 14. Statistical comparison between the two groups was performed using analysis of covariance with adjustment of baseline urinary status as a covariate. Among 80 patients, the total I-PSS was significantly lower in the CIU-SP group than in the C-SP group in the cohort with mild urinary symptoms (P = 0.005), but not in those with moderate/severe symptoms (P = 0.521). The CIU-SP group showed significantly improved I-PSS and OABSS daytime frequencies, with the highest *t* statistic (2.47 and 2.10, respectively) among subdomains of both symptom scores compared with the C-SP group (both P < 0.001). In multivariate regression analysis, the stent placement method (CIU-SP vs. C-SP) was independently associated with the I-PSS daytime frequency on day 14 (P = 0.017). This study suggests that CIU-SP significantly improved stent-related daytime frequency compared with C-SP, and it may benefit especially those patients who have mild urinary symptoms before the placement of ureteral stents.

## Introduction

Ureteral stents are an effective medical device for improving urinary passage obstruction mainly due to ureteral stricture and stone impaction^1^. These stents are also useful for preserving patency in ureteral injury during the wound healing process^2^. In the case of ureteroscopic lithotripsy (URS), ureteral stents are commonly placed with the intention of preventing urinary obstruction due to ureteral edema at the surgical site. This leads to a reduction in the risk of postoperative urinary infection^3^. However, despite such advantages, this process causes ureteral stent discomfort, such as body pain and impairment of urinary symptoms, and affects the patient’s quality of life (QoL)^4^. Most of these symptoms have been considered to be attributed to ureteral spasm or contact of the distal end of the stent on the bladder wall^5^. However, there is no uniform consensus on how to improve stent-related urinary discomfort.

Recently, our research group conducted a prospective, randomized, controlled trial to evaluate the efficacy of complete intra-ureteral stent placement (CIU-SP) vs. conventional stent placement (C-SP) in patients with an indwelling loop type of ureteral stent after URS^6^. The main concept of CIU-SP was omitting the distal end of ureteral stents to reduce irritation on the trigonal area of the bladder. We found that CIU-SP significantly reduced stent-related pain (as a primary outcome measure) and lower urinary tract symptoms (LUTS) (as secondary outcome measures) compared with C-SP on postoperative days 3 and 14. With regard to urinary symptoms, these should be affected not only by URS or the ureteral stent placement procedure, but are also largely affected by baseline LUTS status^7–9^. To appropriately assess the efficacy of CIU-SP in stent-related urinary symptoms To appropriately assess the efficacy of CIU-SP in ameliorating stent-related urinary symptoms and identify the subgroup that could benefit from CIU-SP compared to C-SP, we performed a post-hoc analysis to compare the mean changes in urinary symptom scores from baseline to day 14 between the two stent placement groups, with adjustment of the baseline urinary status as a covariable^10^. Furthermore, we examined the most relevant subdomain in urinary symptom scores that is associated with improvement by CIU-SP vs. C-SP.

## Results

### Patients’ characteristics

Overall, the mean total International Prostate Symptom Score (I-PSS) and Overactive Bladder Symptom Score (OABSS) were 9.57 ± 7.83 (categorized as moderate symptoms^11^) and 3.11 ± 2.31 (categorized as mild symptoms^12^), respectively. Of 80 patients, 41 (51.2%; CIU-SP: 21, C-SP: 20) and 39 (48.8%; CIU-SP: 18, C-SP: 21) had mild and moderate/severe I-PSS, respectively; and 67 (83.8%; CIU-SP: 30, C-SP: 37) had mild symptoms, and 13 (16.2%; CIU-SP: 9, C-SP: 4) had mild and moderate/severe OABSS, respectively. Baseline characteristics between the two groups were well balanced, except for OABSS urgency (P = 0.024; Table 1).

**Table 1:** Baseline patient characteristics of our cohort.

Variable	Overall	Complete intraureteral stent placement group	Conventional stent placement group	P value
	(n = 80)	(n = 39)	(n = 41)	
Age, years	58.41 ± 13.37	60.92 ± 14.22	56.02 ± 12.21	0.102
**Sex**				0.659
Female	28 (35.0)	15 (38.5)	13 (31.7)	
Male	52 (65.0)	24 (61.5)	28 (68.3)	
Body mass index, kg/m^2^	25.20 ± 4.18	25.23 ± 3.86	25.17 ± 4.51	0.953
**Stone location**				0.359
Renal pelvis	29 (36.2)	12 (30.8)	17 (41.5)	
Ureter	51 (63.7)	27 (69.2)	24 (58.5)	
**I-PSS**				
Total score	9.57 ± 7.94	8.82 ± 7.83	10.29 ± 8.07	0.410
Q1. Incomplete emptying	0.97 ± 1.40	0.72 ± 1.07	1.22 ± 1.62	0.109
Q2. Daytime frequency	1.50 ± 1.47	1.26 ± 1.55	1.73 ± 1.36	0.149
Q3. Intermittency	0.54 ± 1.17	0.46 ± 1.10	0.61 ± 1.24	0.574
Q4. Urgency	0.69 ± 1.18	0.95 ± 1.43	0.44 ± 0.81	0.052
Q5. Weak stream	1.00 ± 1.41	0.95 ± 1.41	1.05 ± 1.41	0.752
Q6. Straining	0.38 ± 1.04	0.26 ± 0.88	0.49 ± 1.16	0.321
Q7. Nocturia	1.61 ± 1.32	1.67 ± 1.36	1.56 ± 1.29	0.722
Voiding symptom subscore (Q1 + 3 + 5 + 6)	2.89 ± 4.17	2.38 ± 3.89	3.37 ± 4.42	0.296
Storage symptom subscore (Q2 + 4 + 7)	3.80 ± 3.07	3.87 ± 3.55	3.73 ± 2.58	0.840
Quality of life index	2.89 ± 1.90	2.56 ± 1.70	3.20 ± 2.04	0.138
**OABSS**				
Total score	3.11 ± 2.33	3.49 ± 2.61	2.76 ± 2.00	0.166
Q1. Daytime frequency	0.62 ± 0.56	0.54 ± 0.55	0.71 ± 0.56	0.179
Q2. Nighttime frequency	1.38 ± 1.02	1.44 ± 1.05	1.32 ± 1.01	0.607
Q3. Urgency	0.88 ± 1.18	1.18 ± 1.23	0.59 ± 1.07	0.024
Q4. Urgency incontinence	0.24 ± 0.66	0.33 ± 0.87	0.15 ± 0.36	0.208

Non- was used for statistical analysis.

Data are presented as number (%) or mean ± standard deviation.

*I-PSS* International Prostate Symptom Score, *OABSS* Overactive Bladder Symptom Score.

### Changes in urinary symptom scores from baseline to day 14 due to stent placement

**Figure 1:**
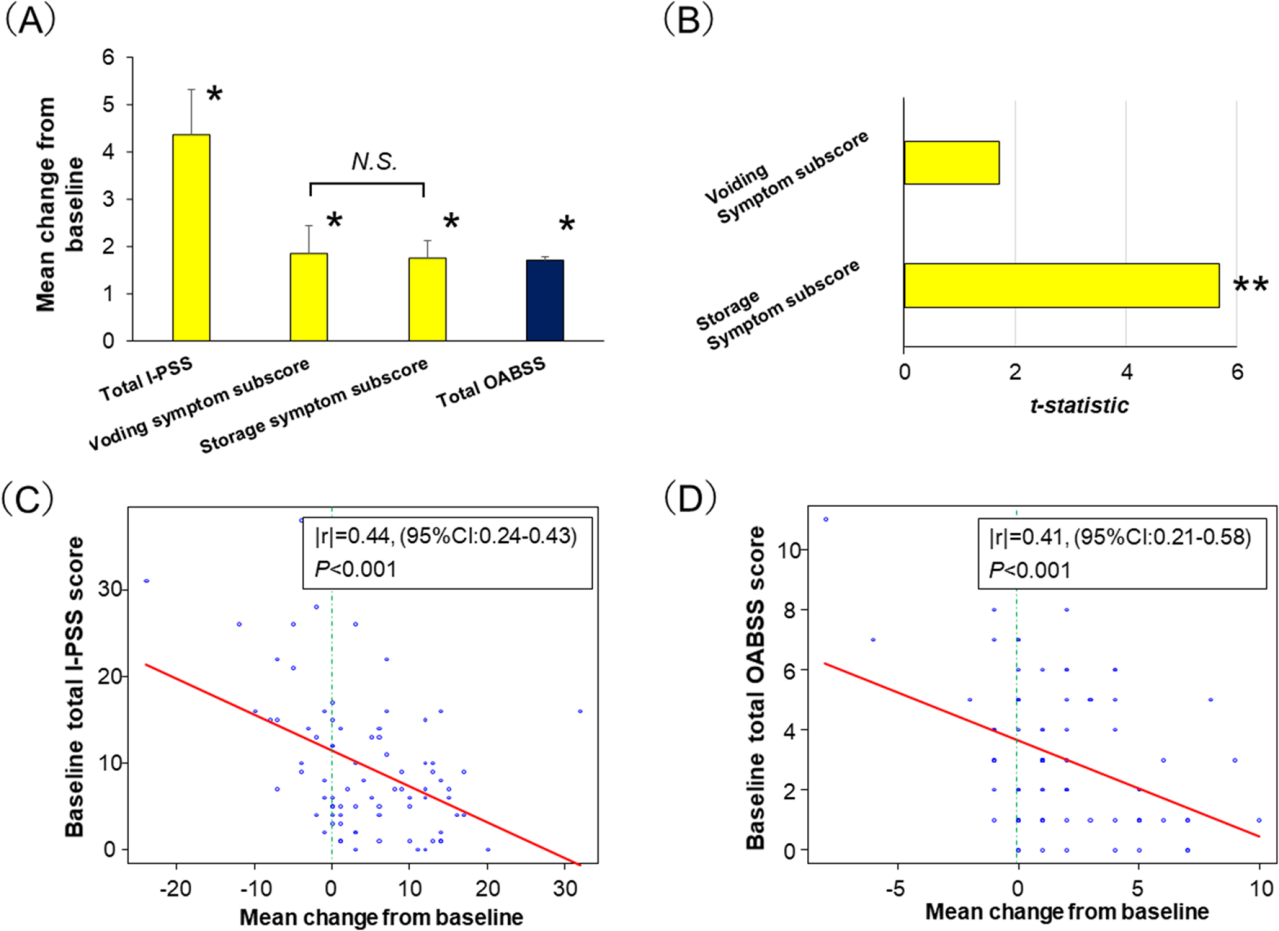
(**A**) Mean ± standard error changes from baseline to day 14 in all patients. *P < 0.05 (day 14 vs. baseline) using the paired *t *test. *N.S.* no significant difference using the unpaired *t* test. (**B**) Correlation analysis between International Prostate Symptom Score (I-PSS) voiding/storage symptom subscores and the I-PSS quality of life (QoL) index. ***P < 0.001 using multiple linear regression analysis. Explanatory variables: I-PSS voiding and storage symptom subscores (continuous); response variable: I-PSS QoL index (continuous). Correlation analysis between baseline total urinary symptom scores and mean change in total urinary symptom scores from the baseline; (**C**) I-PSS and (**D**) OABSS. The correlations between data were evaluated using Spearman’s rank correlation test.

The mean changes in the I-PSS and OABSS in all patients are shown in Fig. 1 and Table 2. The total mean I-PSS, I-PSS voiding symptom subscore (Q1 + Q3 + Q5 + Q6), I-PSS storage symptom subscore (Q2 + Q4 + Q7), and total OABSS on day 14 were significantly increased compared with baseline (all P < 0.05; Fig. 1A). Although there was no significant difference between the voiding and storage symptom subscores (P = 0.884, Fig. 1A and Table 2), the storage symptom subscore was independently associated with deterioration of the I-PSS QoL index due to stent placement (β = 0.382, standard error [SE] = 0.042, *t* statistic = 5.682, P < 0.001; Fig. 1B). Analysis of the correlation between baseline symptom scores and the mean change in scores from the baseline revealed that lower baseline scores were significantly correlated with worse urinary symptom outcome in both I-PSS (|r| = 0.44, P < 0.001; Fig. 1C) and OABSS (|r| = 0.41, P < 0.001; Fig. 1D).

**Table 2:** Mean change in each parameter from baseline to postoperative day 14 between.

Variable	Overall	Complete intraureteral stent placement group	Conventional stent placement group	P value
	(n = 80)	(n = 39)	(n = 41)	
**I-PSS**				
Q1. Incomplete emptying	1.16 ± 1.77	1.00 ± 0.24	1.31 ± 0.30	0.095
Q2. Daytime frequency	0.78 ± 1.63	0.56 ± 0.23	0.97 ± 0.27	0.048
Q3. Intermittency	0.64 ± 3.59	0.25 ± 0.21	1.00 ± 0.75	0.346
Q4. Urgency	0.62 ± 1.66	0.25 ± 0.23	0.97 ± 0.27	0.245
Q5. Weak stream	0.06 ± 1.38	0.15 ± 0.21	0.26 ± 0.22	0.082
Q6. Straining	0.00 ± 0.91	0.18 ± 0.11	-0.17 ± 0.16	–^a^
Q7. Nocturia	0.36 ± 0.97	0.23 ± 0.13	0.48 ± 0.16	0.242
Voiding symptom subscore (Q1 + 3 + 5 + 6)	1.86 ± 5.16	1.28 ± 0.57	2.41 ± 0.98	0.172
Storage symptom subscore (Q2 + 4 + 7)	1.76 ± 3.29	1.05 ± 0.46	2.43 ± 0.54	0.038
QOL index	0.74 ± 2.33	0.51 ± 0.35	0.95 ± 0.37	0.040
**OABSS**				
Q1. Daytime frequency	0.19 ± 0.68	0.12 ± 0.08	0.24 ± 0.12	0.087
Q2. Nighttime frequency	0.42 ± 0.79	0.33 ± 0.09	0.51 ± 0.14	0.392
Q3. Urgency	0.84 ± 1.69	0.48 ± 0.23	1.17 ± 0.28	0.302
Q4. Urgency incontinence	0.25 ± 0.99	0.17 ± 0.16	0.31 ± 0.14	–^a^

Analysis of covariance was used for statistical analysis.

Data are presented as mean ± standard deviation.

*I-PSS* International Prostate Symptom Score, *OABSS* Overactive Bladder Symptom Score.

^a^Not calculated due to significant interaction between group variables and covariates.

### Comparison of changes in urinary symptom scores from baseline to day 14 between CIU-SP and C-SP using analysis of covariance (ANCOVA) with adjustment of baseline urinary status

**Figure 2:**
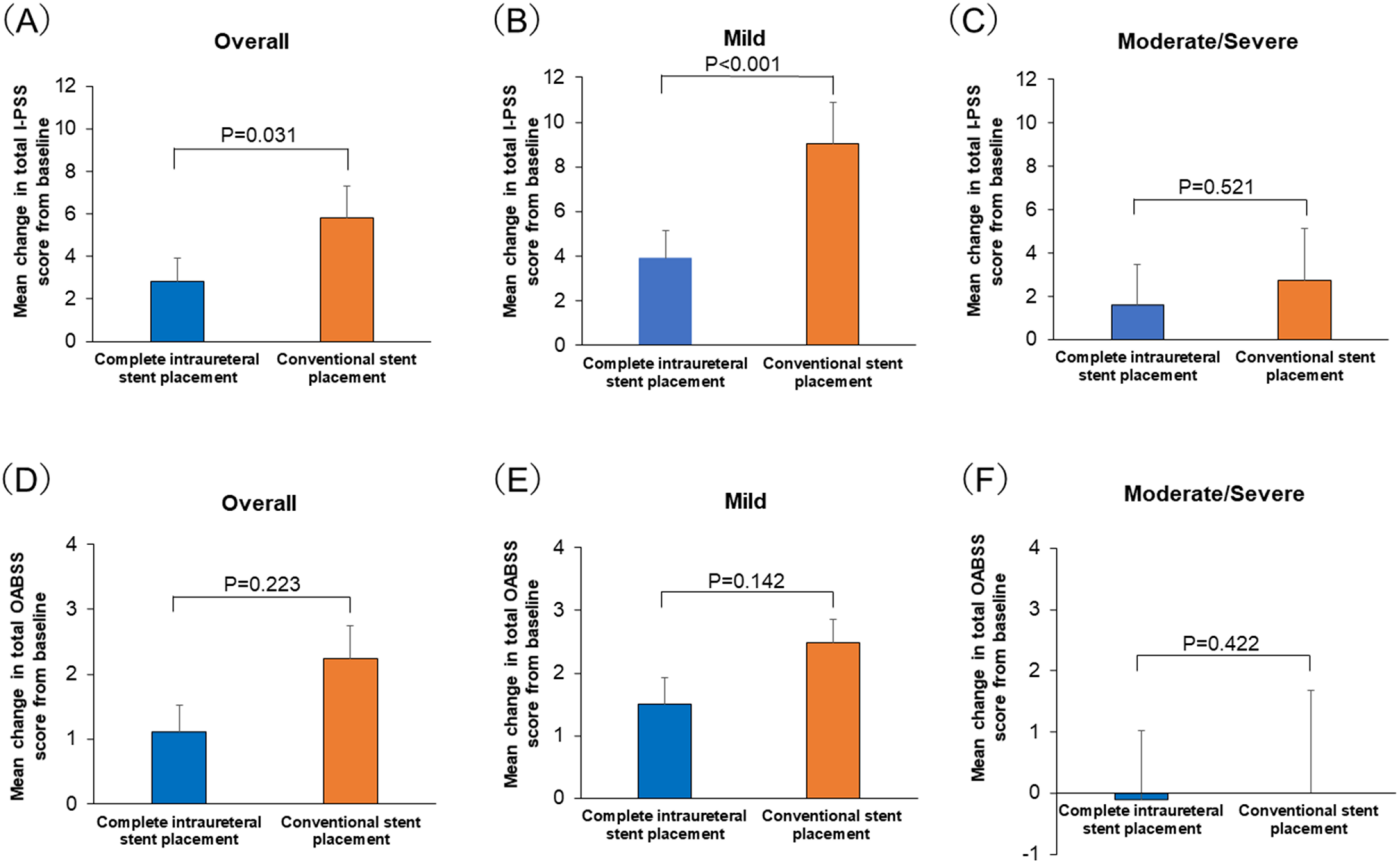
Mean ± standard error changes in the total urinary symptom scores, total International Prostate Symptom Score (IPSS; **A**‒**C**), and Overactive Bladder Symptom Score (OABSS; **D**‒**F**), from baseline to day 14. (**A**) All patients, (**B**) patients with mild symptoms, (**C**) patients with moderate/severe symptoms, (**D**) all patients, (**E**) patients with mild symptoms, and (**F**) patients with moderate/severe symptoms. Analysis of covariance was used for statistical analysis of complete intraureteral stent placement vs. conventional stent placement. *P < 0.05, *N.S.* no significant difference.

The mean total I-PSS was significantly lower in the CIU-SP group than in the C-SP group in the overall patient population (2.84 ± 1.09 vs. 5.80 ± 1.50, P = 0.031; Fig. 2A) and in patients with mild symptoms (3.90 ± 5.58 vs. 9.05 ± 6.49, P < 0.001; Fig. 2B), but not in patients with moderate/severe symptoms (Fig. 2C). There was no significant difference in the total OABSS between the groups in the overall patient population and in the subcategories of patients (all, P > 0.05; Fig. 2D‒F). With regard to the subscores, the I-PSS daytime frequency (0.56 ± 0.23 vs. 0.97 ± 0.27, P = 0.049), I-PSS storage symptom subscore (1.05 ± 0.46 vs. 2.43 ± 0.54, P = 0.038), and I-PSS QoL index (0.51 ± 0.35 vs. 0.95 ± 0.37, P = 0.040) were significantly lower in the CIU-SP group than in the C-SP group (Table 2). Although the OABSS daytime frequency tended to be lower in the CIU-SP group than in the C-SP group (0.12 ± 0.08 vs. 0.24 ± 0.12, P = 0.087), there were no significant differences in the OABSS subscores between the groups (Table 2).

### Correlations between the stent placement method and urinary symptom subscores on day 14

**Figure 3:**
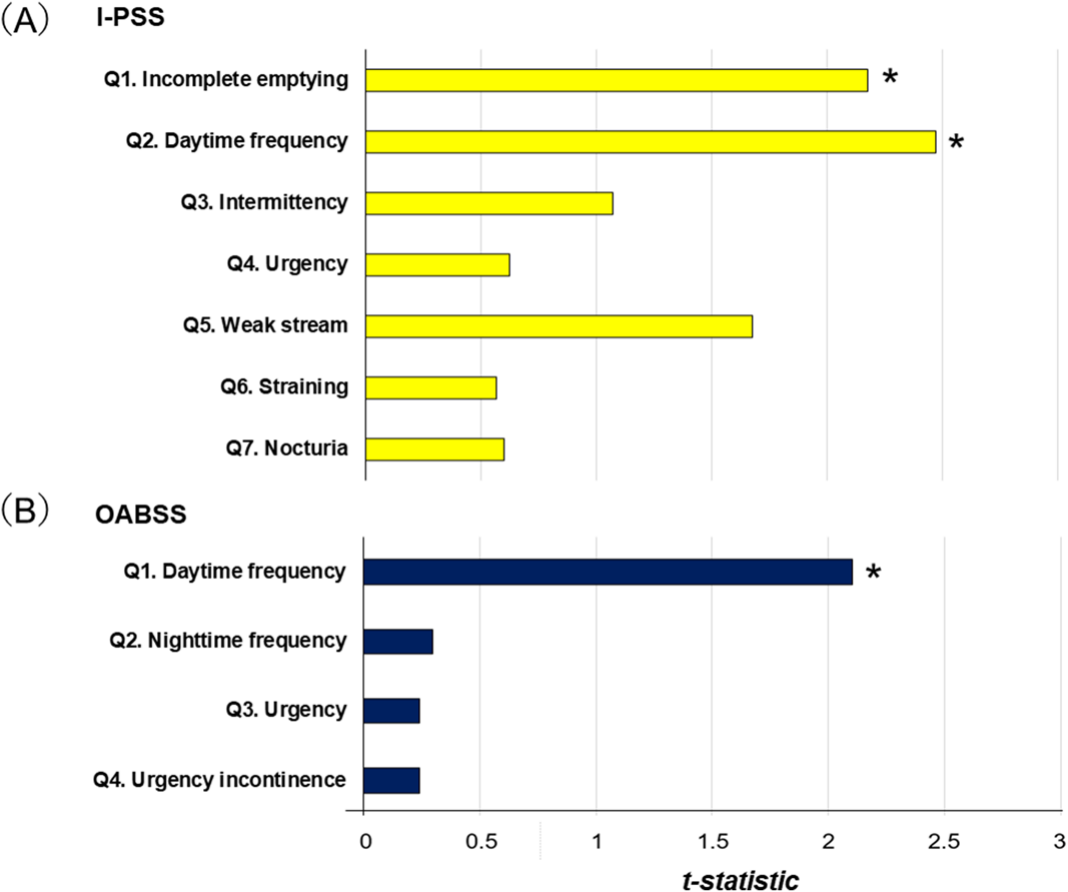
Correlations between stent placement methods and urinary symptom subscores on postoperative day 14. (**A**) International Prostate Symptom Score (IPSS) and (**B**) overactive Bladder Symptom Score (OABSS). *P < 0.05 using simple linear regression analysis. Explanatory variable: stent placement method (category: complete intraureteral stent placement vs. conventional stent placement); response variable: each urinary symptom subdomain (continuous).

To identify the subdomain of the I-PSS or OABSS that was the most strongly associated with the stent placement method (CIU-SP vs. C-SP), simple linear regression analysis was performed. The I-PSS incomplete emptying (SE = 0.38, *t* statistic = 2.18, P < 0.001), I-PSS daytime frequency (β = 0.89, SE = 0.38, *t* statistic = 2.47, P < 0.001), and OABSS daytime frequency (β = 0.28, SE = 0.14, *t* statistic = 2.10, P = 0.039) were significantly correlated with the stent placement method (Fig. 3). Daytime frequency showed the highest *t* statistic among the I-PSS and OABSS subdomains (2.47 and 2.10, respectively) (Fig. 3).

### Multivariate analysis of clinical factors associated with daytime frequency of the I-PSS or OABSS on day 14

According to the results from simple regression analysis described above, multiple regression analysis was performed to investigate the relationships between potential clinical variables and I-PSS/OABSS daytime frequency. Body mass index (β =  − 0.160, SE = 0.045, *t* statistic =  − 3.456, P = 5.859 × 10^−4^) and stent placement method (β = 0.853, SE = 0.349, *t* statistic = 2.402, P = 0.017) were significantly associated with the I-PSS daytime frequency (Table 3). No factors were significantly correlated with the OABSS daytime frequency, with only a tendency for the stent placement method (β = 0.257, SE = 0.141, *t* statistic = 1.859, P = 0.072) (Table 3).

**Table 3:** Multivariate analysis assessing the association between characteristic parameters and I-PSS- or OABSS-daytime frequency on postoperative day 14.

	β	SE	*t* statistic	P value
**I-PSS daytime frequency**				
Age, years	− 0.007	0.014	− 0.504	0.616
Sex (female vs. male)	0.120	0.377	0.320	0.750
Body mass index, kg/m^2^	− 0.152	0.044	− 3.456	< 0.001
Stone location (renal pelvis vs. ureter)	0.033	0.372	0.088	0.930
Stent placement method (CIU-SP vs. C-SP)	0.840	0.350	2.402	0.019
**OABSS daytime frequency**				
Age, years	− 0.003	0.006	− 0.463	0.543
Sex (female vs. male)	− 0.025	0.152	− 0.162	0.930
Body mass index, kg/m^2^	− 0.023	0.018	− 1.316	0.192
Stone location (renal pelvis vs. ureter)	− 0.093	0.141	− 0.618	0.539
Stent placement method (CIU-SP vs. C-SP)	0.262	0.141	1.859	0.067

Multiple linear regression model was used for statistical analysis.

*SE* standard error, *CIU-SP* complete intraureteral stent placement, *C-SP* conventional stent placement, *I-PSS* International Prostate Symptom Score, *OABSS* Overactive Bladder Symptom Score.

## Discussion

In the present study, we focused on stent-related urinary symptoms in patients with an indwelling ureteral stent after URS. The I-PSS storage symptom subscore was correlated with the I-PSS QoL index rather than its voiding symptom subscore. A novel placement technique, CIU-SP, was significantly superior to C-SP in terms of improving the total I-PSS score, daytime frequency, storage symptom subscore, and QoL index. Patients having mild urinary symptoms at the baseline could more benefit from CIU-SP than from C-SP. Daytime frequency in the I-PSS and OABSS was the most relevant subdomain that was improved by CIU-SP compared with C-SP. Finally, the stent placement method (CIU-SP vs. C-SP) was an independent clinical factor for predicting improvement of the I-PSS daytime frequency.

In contrast to our previous report^6^, this post-hoc analysis had several strengths for accurately evaluating stent-related LUTS for the following reasons: (1) measuring the mean change in urinary symptoms from baseline to day 14; (2) applying analysis of covariance for adjusting baseline covariates; (3) selecting day 14 as the final evaluation date for minimizing the confounding effect of discomfort of the URS procedure^13^; and (4) examining details regarding stent-related LUTS using the validated LUTS-specific indicators^11,12^. Therefore, our results have added new evidence on management of improving stent-related urinary discomfort and QoL.

In the field of LUTS research, storage-related symptoms are the most clinically bothersome and affect patients’ QoL in those with benign prostatic hyperplasia (BPH)/LUTS or overactive bladder (OAB)^14–17^. Charles et al. found that nocturia and daytime frequency were the primary and secondary chief complaints, respectively, based on the American Urological Association Symptom Index in 1240 men with BPH^15^. McVary et al. suggested that the symptom of bother and QoL were affected two-fold by storage I-PSS questions (Q2, Q4, Q7) vs. questions on voiding symptoms (Q1, Q3, Q5, Q6)^16^. OAB, namely a storage symptom disorder, affected patients’ mental health, work productivity, and health-related QoL, regardless of sex^17^.

In the present study, we found that patients’ QoL was affected approximately three-fold by a storage symptom compared with a voiding symptom, despite the fact that both symptoms equally occurred because of stent placement. Similar to the approach for BPH/LUTS or OAB, management of storage symptoms is important in controlling ureteral stent-related urinary discomfort. Physical or chemical stimulation on the bladder wall causes release of chemicals, including ATP, acetylcholine, prostaglandins, and nitric oxide, which modulate the activity of either afferent nerves or muscular components of the bladder wall^18^. According to such underlying mechanism(s), CIU-SP may be a reasonable placement method because of not presenting the distal end of the stent. This can prevent iatrogenic physical irritation on the bladder wall. Notably, in our study, CIU-SP significantly reduced daytime frequency among urinary symptom subdomains compared with C-SP, whereas the rate of nocturia was equal in both groups. Based on the mechanism(s) mentioned above, these findings are easy to understand because the absence of a distal tip of the stent might reduce physical stimulus on the bladder during daytime activities.

Furthermore, we also found a negative correlation between the degree of the urinary status at baseline and the change in urinary symptoms after ureteral stent placement. Indeed, patients who had mild urinary symptoms could benefit from CIU-SP, whereas those with moderate/severe symptoms showed little impact, regardless of placement techniques. Thus, the clinical significance of CIU-SP demonstrated in our previous study^6^ could be attributed to the inclusion of more than half of the patients with favorable urinary symptoms before the URS surgery in the study. In other words, patients who have moderate or more urinary symptoms could tolerate even conventional stent placement. This information can be applied in daily clinical practice as well as in clinical trial protocols associated with ureteral stent placement.

To date, many studies have attempted to alleviate stent-related discomfort by agents, such as alpha-1 blockers, anticholinergics, and beta-3 adrenergic receptor agonists^13,19–22^. These results regarding improvement of urinary symptoms are conflicting, and negative results have been found in several randomized, controlled trials^13,20–22^. We believe that trials using medication should consider not only improvement of stent-related irritation, but also urinary symptoms that the patient has^23^. Nevertheless, most ureteral stent studies did not take into account the change from baseline or baseline LUTS status^13,19–22^. Therefore, actual results of previous studies may change if considering these factors for statistical analysis. Unlike trials of medications, we focused on examining the pure effect of stent placement methods on urinary symptoms, and found that CIU-SP was a better placement technique than C-SP. Unfortunately, even when using CIU-SP, some patients still suffer from stent-related urinary symptoms. Therefore, further investigations are required to determine the mechanism(s) that are associated with these symptoms (i.e., extraction string, intravesical inflammation, or irradiation pain), for providing a more comfortable method for patients who require ureteral stent placement.

We acknowledge several limitations that should be interpreted with caution. First, this post-hoc study was not intended to be designed when performing the primary study. Therefore, there was a lack of calculation of required sample size for this study. Second, we used only the loop type of stents instead of double j stents, which are commonly used. Although this study could not determine the best management for using double j stents, we believe that our findings could be useful for developing a new stent design that can reduce irritation of the bladder wall. Finally, as we previously mentioned^6^, we did not assess the safety of CIU-SP in patients with distal ureteral stones. Therefore, further studies including patients with distal ureteral stones are required in the future.

## Conclusion

This study highlights the asymmetrical relationship between ureteral stent-related storage and voiding LUTS for patients’ urinary QoL. CIU-SP may be a more beneficial strategy for patients who have mild urinary symptoms at baseline than for those who have moderate/severe symptoms. Furthermore, CIU-SP might help reduce ureteral stent-related storage symptoms compared with C-SP, especially in terms of improving daytime frequency.

## Methods

### Participants and study design

This was a post-hoc analysis that used the same data of a previous prospective, single-blind, randomized, clinical trial, which was registered at the University Hospital Medical Information Network (UMIN00017067)^6^. This study was approved by the ethics board of Kansai Medical University (IRB No. 2016503), and all patients provided written informed consent. This trial strictly followed the 2010 Consolidated Standards of Reporting Trials (CONSORT) statement guidelines^24^. Details of this trial, and inclusion and exclusion criteria were described previously^6^. Briefly, patients who were aged > 20 years who underwent unilateral URS with planned ureteral stent insertion were included. However, patients who had concomitant use of alpha-1 blockers, anticholinergics, corticosteroids, calcium channel blockers, and analgesics, distal ureteral stones, and preoperative ureteral stenting were excluded. Patients were equally randomized in a 1:1 ratio into the CIU-SP and C-SP groups. The modified intention-to-treat population, except for those without follow-up, sufficient clinical data, or those who withdrew consent after randomization, was used for analysis (Supplementary Figure 1).

### Intervention

For all patients, the I-PSS (mild: ≤ 7, moderate: 8–19, severe: ≥ 20)^11^ and the OABSS (mild: ≤ 5, moderate: 6–11, severe: ≥ 12)^12^ at baseline and day 14 were obtained (Supplementary Figure 1). The details of surgical intervention of this study were also described previously^6^. Briefly, all patients underwent unilateral URS under spinal anesthesia with or without a ureteral access sheath. After URS, a Polaris Loop Ureteral Stent (Boston Scientific, Malborough, MA, USA) with a string was inserted by the two placement methods (i.e., CIU-SP and C-SP) according to the actual ureteral length. Finally, the stent string was cut approximately 10 cm from the tip of the urethra after insertion of the urethral catheter. For discharge medication, only oral diclofenac sodium 25 mg was allowed to be used during the study period (use of antimuscarinics or alpha-blockers were prohibited). On day 14, the ureteral stent was removed at the outpatient clinic with the extraction string.

### Endpoints

The primary endpoint was the mean change in the total I-PSS and OABSS from baseline to day 14. The secondary endpoints were the mean change in the I-PSS and OABSS subscores from baseline to day 14. These analyses were not part of the protocol-specified primary or secondary endpoints of the previous study^6^.

### Statistical analysis

Continuous data are expressed as mean ± standard deviation. The Chi-square test was used to compare nominal variables, and the paired or non-paired *t* test was used to compare continuous variables. The correlations between data were evaluated using Spearman’s rank correlation test. For analysis of endpoints, ANCOVA was applied for comparison between the two groups (the placement group as a factor and baseline as a covariate). Simple or multiple linear regression analysis was applied to assess the association between the urinary symptom scores and potential factors, with 15 subjects per variable as the minimum required sample size^25^. The *t* statistic in linear regression analysis, which was calculated as the ratio of an estimated coefficient (β) to its standard error, was used to test the hypothesis that a coefficient is equal to zero. All statistical analyses were performed using EZR version 1.37 (Saitama Medical Center, Jichi, Japan)^26^. A two-sided P value of < 0.05 was considered statistically significant.

## Supplementary information

Supplementary Figure 1.

## Abbreviations

BPH: Benign prostatic hyperplasia
C-SP: Conventional stent placement
CIU-SP: Complete intraureteral stent placement
I-PSS: International Prostate Symptom Score
LUTS: Lower urinary tract symptoms
OABSS: Overactive Bladder Symptom Score
QoL: Quality of life
SE: Standard error
URS: Ureteroscopy

## References

[1] Nabi G, Cook J, N'Dow J, McClinton S (2007). Outcomes of stenting after uncomplicated ureteroscopy: systematic review and meta-analysis. BMJ.

[2] Burks FN, Santucci RA (2014). Management of iatrogenic ureteral injury. Ther. Adv. Urol..

[3] Ordonez M, Hwang EC, Borofsky M, Bakker CJ, Gandhi S, Dahm P (2019). Ureteral stent versus no ureteral stent for ureteroscopy in the management of renal and ureteral calculi. Cochrane Database Syst. Rev..

[4] Scarneciu I, Lupu S, Pricop C, Scarneciu C (2015). Morbidity and impact on quality of life in patients with indwelling ureteral stents: a 10-year clinical experience. Pak. J. Med. Sci..

[5] Joshi HB, Newns N, Stainthorpe A, MacDonagh RP, Keeley FX, Timoney AG (2003). Ureteral stent symptom questionnaire: development and validation of a multidimensional quality of life measure. J. Urol..

[6] Yoshida T, Inoue T, Taguchi M (2019). Efficacy and safety of complete intraureteral stent placement versus conventional stent placement in relieving ureteral stent related symptoms: a randomized, prospective, single blind, multicenter clinical trial. J. Urol..

[7] Nunes EV, Pavlicova M, Hu MC (2011). Baseline matters: the importance of covariation for baseline severity in the analysis of clinical trials. Am. J. Drug Alcohol Abuse.

[8] Hernández AV, Steyerberg EW, Taylor GS, Marmarou A, Habbema JD, Maas AI (2005). Subgroup analysis and covariate adjustment in randomized clinical trials of traumatic brain injury: a systematic review. Neurosurgery.

[9] Kaplan SA, He W, Koltun WD, Cummings J, Schneider T, Fakhoury A (2013). Solifenacin plus tamsulosin combination treatment in men with lower urinary tract symptoms and bladder outlet obstruction: a randomized controlled trial. Eur. Urol..

[10] Colantuoni E, Rosenblum M (2015). Leveraging prognostic baseline variables to gain precision in randomized trials. Stat. Med..

[11] Barry MJ, Fowler FJ, O'Leary MP (1992). The American Urological Association symptom index for benign prostatic hyperplasia. The Measurement Committee of the American Urological Association. J. Urol..

[12] Homma Y, Gotoh M (2009). Symptom severity and patient perceptions in overactive bladder: how are they related?. BJU Int..

[13] Beddingfield R, Pedro RN, Hinck B, Kreidberg C, Feia K, Monga M (2009). Alfuzosin to relieve ureteral stent discomfort: a prospective, randomized, placebo controlled study. J. Urol..

[14] Agarwal A, Eryuzlu LN, Cartwright R (2014). What is the most bothersome lower urinary tract symptom? Individual- and population-level perspectives for both men and women. Eur. Urol..

[15] Welliver C, Sulaver R, Whittington A (2015). Analyzing why men seek treatment for lower urinary tract symptoms and factors associated with nonimprovement. Urology.

[16] McVary KT, Peterson A, Donatucci CF (2016). Use of structural equation modeling to demonstrate the differential impact of storage and voiding lower urinary tract symptoms on symptom bother and quality of life during treatment for lower urinary tract symptoms associated with benign prostatic hyperplasia. J. Urol..

[17] Coyne KS, Sexton CC, Kopp ZS, Ebel-Bitoun C, Milsom I, Chapple C (2011). The impact of overactive bladder on mental health, work productivity and health-related quality of life in the UK and Sweden: results from EpiLUTS. BJU Int..

[18] Fry CH, Vahabi B (2016). The role of the mucosa in normal and abnormal bladder function. Basic Clin. Pharmacol. Toxicol..

[19] Lamb AD, Vowler SL, Johnston R, Dunn N, Wiseman OJ (2011). Meta-analysis showing the beneficial effect of α-blockers on ureteric stent discomfort. BJU Int..

[20] Norris RD, Sur RL, Springhart WP (2008). A prospective, randomized, double-blinded placebo-controlled comparison of extended release oxybutynin versus phenazopyridine for the management of postoperative ureteral stent discomfort. Urology.

[21] Oh JJ, Lee S, Cho SY (2017). Effects of naftopidil on double-J stent-related discomfort: a multicenter, randomized, double-blinded, placebo-controlled study. Sci. Rep..

[22] Tae BS, Cho S, Jeon BJ (2018). Does mirabegron relieve ureteric stent-related discomfort? A prospective, randomized, multicentre study. BJU Int..

[23] Kozminski MA, Wei JT, Nelson J, Kent DM (2015). Baseline characteristics predict risk of progression and response to combined medical therapy for benign prostatic hyperplasia (BPH). BJU Int..

[24] Moher D, Hopewell S, Schulz KF (2012). CONSORT 2010 explanation and elaboration: updated guidelines for reporting parallel group randomised trials. Int. J. Surg..

[25] Schmidt FL (1971). The relative efficiency of regression and simple unit predictor weights in applied differential psychology. Educ. Psychol. Meas..

[26] Kanda Y (2013). Investigation of the freely available easy-to-use software 'EZR' for medical statistics. Bone Marrow Transpl..

