# Supplementary material for: Complete intraureteral stent placement relieves daytime urinary frequency compared with conventional placement in patients with an indwelling ureteral stent: post-hoc analysis of a randomized, controlled trial
Source: Sci Rep. 2020 Sep 28;10:15892. doi: 10.1038/s41598-020-72937-0 (PMC7522210; doi:10.1038/s41598-020-72937-0)
Supplement: Supplementary file 1 — Supplementary Figure 1. [file 41598_2020_72937_MOESM1_ESM.pdf]

Complete Intraureteral Stent Placement Relieves Daytime Urinary Frequency Compared with Conventional Placement in Patients with an Indwelling Ureteral Stent: Post-hoc Analysis of a Randomized, Controlled Trial

Tomoaki Matsuzaki[1†], Takashi Yoshida[1,2†], Takashi Murota[1,3], Kazuyoshi Nakao[1], Makoto Taguchi[1], Hidefumi Kinoshita[1], and Tadashi Matsuda[1]

[1]Department of Urology and Andrology, Kansai Medical University, Osaka, Japan
[2]Department of Urology and Andrology, Kori Hospital, Kansai Medical University, Osaka, Japan
[3]Department of Urology and Andrology, General Medical Center, Kansai Medical University, Osaka, Japan

[†]These authors contributed equally to the work

Corresponding Author: Takashi Yoshida, MD
Department of Urology and Andrology, Kansai Medical University, 2-3-1 Shinmachi, Hirakata, Osaka 573-1191, Japan
Tel: +81-72-804-0101; fax: +81-72-804-2089
E-mail: yoshidtk@takii.kmu.ac.jp

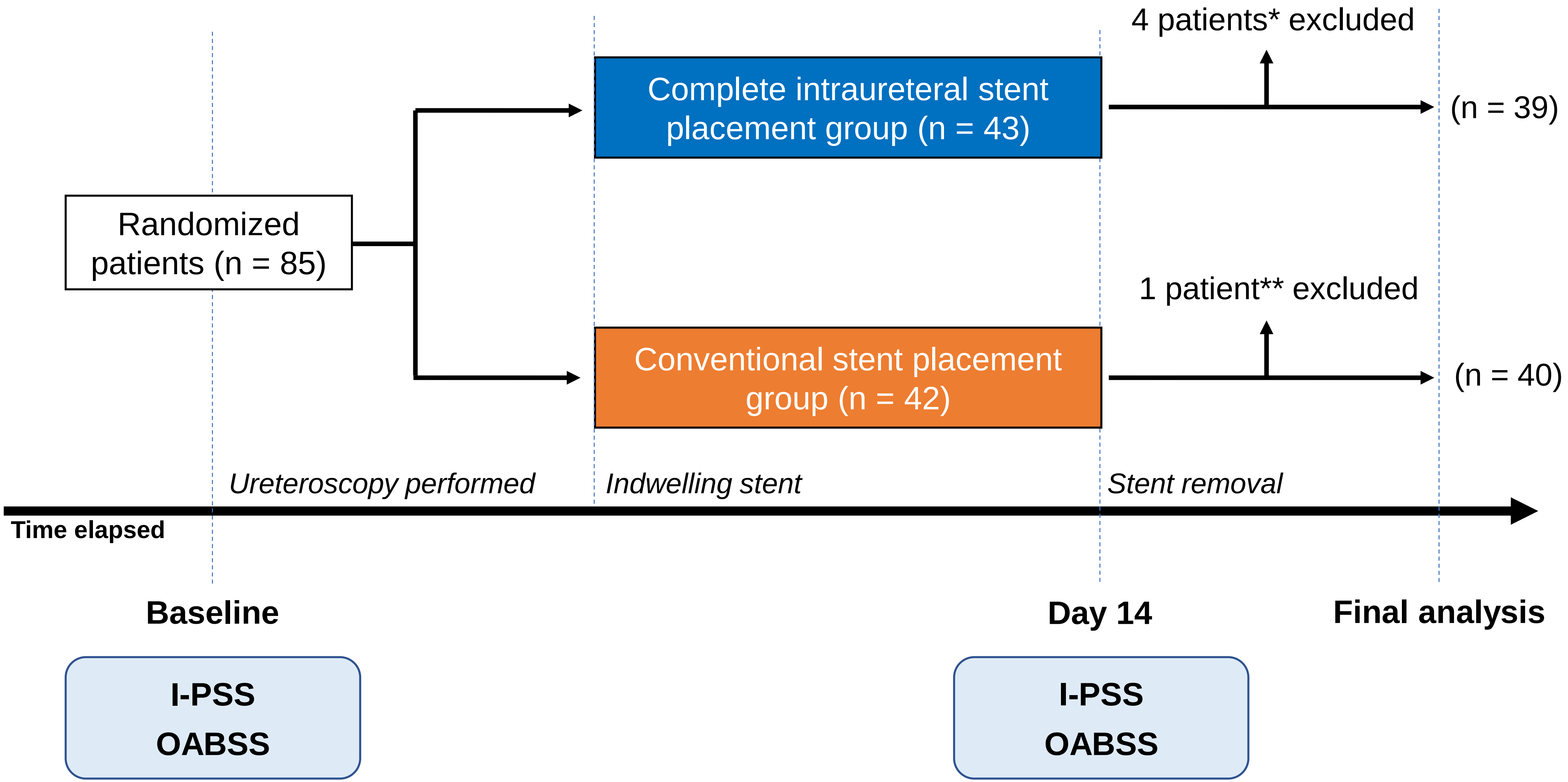

Supplementary Figure 1. Study design of the post-hoc analysis. *Lost to follow-up (n=1), withdrew consent (n=2), and insufficient clinical data (n=1). **Lost to follow-up (n=1). I-PSS, International Prostate Symptom Score; and OABSS, Overactive Bladder Symptom Score.
